# Incidental Vascular Lesion in Explanted Liver: A Rare Case Report and Review of the Literature

**DOI:** 10.1155/2023/8864977

**Published:** 2023-11-06

**Authors:** Rossana Kazemimood, Shohreh Eliaszadeh, Kenneth Wind

**Affiliations:** ^1^The University of Texas Health Science Center at Houston, Houston, TX, USA; ^2^The Scarborough Hospital, Toronto, ON, Canada; ^3^Froedtert South Hospital, Pleasant Prairie, WI, USA

## Abstract

Hepatic small vessel neoplasm (HSVN) is a rare vascular hepatic lesion that is usually an asymptomatic incidental finding. Here we present a case of a 66-year-old male with HSVN who was discovered to have a lesion presenting as a small nodule in an explanted liver. HSVN is a recently described hepatic vascular lesion that has been previously underdiagnosed. It has an uncertain long-term malignant potential, so close follow-up is recommended.

## 1. Introduction

Hepatic vascular tumors demonstrate a range of appearances, from benign cavernous hemangioma to malignant angiosarcoma, the most common primary sarcoma of the liver. The differentiation between these two vascular lesions can usually be performed on routine morphology; however, recently, a few vascular lesions have been reported with features of both categories. The hepatic small vessel neoplasm (HSVN) was first described by Gill et al. [1] in 2016; it has indeterminate features of malignancy and can therefore present a challenging morphologic diagnosis. It is an asymptomatic vascular neoplasm without specific radiographic findings. Thus, a liver biopsy is usually indicated for diagnosis [1].

## 2. Case Report

Our case is a 66-year-old male with a clinical history of alcoholic cirrhosis, diabetes mellitus, hypertension, coronary artery disease, and end-stage renal disease. Before the hepatic explantation, the MRI and CT scans showed no suspicious liver lesions besides cirrhosis.

The patient underwent native liver hepatectomy for a liver transplant. A gross examination of the explanted liver showed one small tan-brown nodule measuring 0.7 cm in the greatest dimension in a background of cirrhotic nodules. No other suspicious lesions were identified.

**Figure 1:**
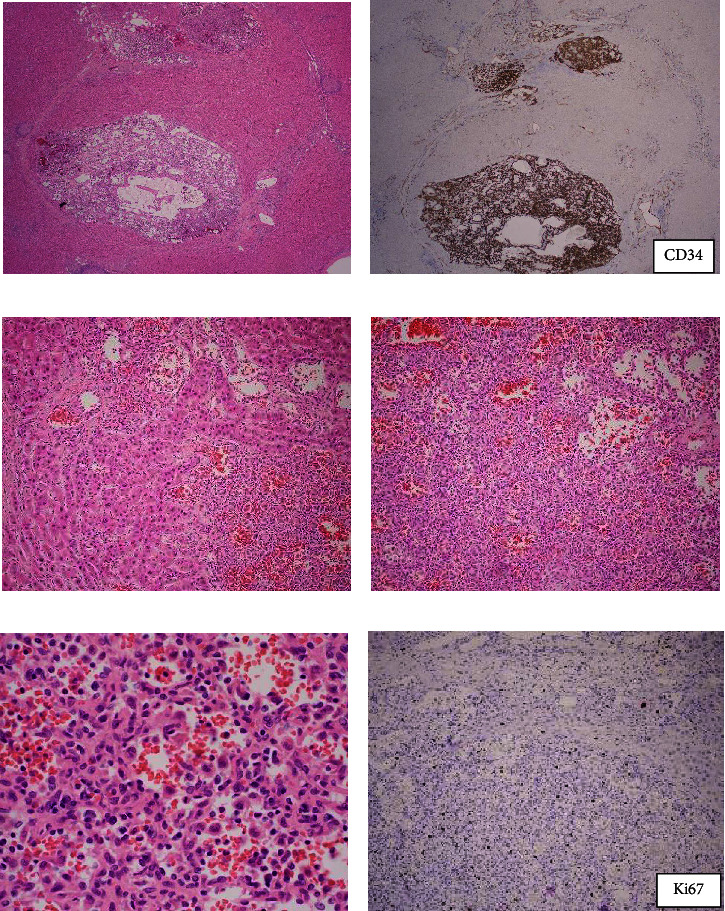
(a) Hepatic small vessel neoplasm (HSVN) (H&E stain, 2x magnification). (b) CD34 (IHC stain, 2x magnification). (c, d) Hepatic small vessel neoplasm (HSVN) with infiltration into sinusoids (H&E stain, 10x magnification). (e) Vascular space with flat to mildly enlarged endothelial cells (H&E stain, 20x magnification). (f) Ki-67 (IHC stain, 10x magnification).

Histologic examination showed vascular lesion, and immunohistochemical stain for CD34 highlights the vessels (Figures 1(a) and 1(b)). The lesion infiltrated into hepatic parenchyma (sinusoids) and was hypercellular (Figures 1(c) and 1(d)). The endothelial cells were mildly enlarged (Figure 1(e)).

The immunohistochemical stain for p53 was wild type. The Ki-67 is about 3% (Figure 1(f)), higher than expected in a hemangioma but lower than expected in an angiosarcoma, which would be at least 10%. A specialist in gastrointestinal and hepatic pathology confirmed the diagnosis of HSVN.

## 3. Discussion

Since 2016 when HSVN was first described [1], more than 25 cases have been published in the literature [2, 3]. This lesion is asymptomatic and usually is an incidental finding. There are no characteristic radiographic findings, and a liver biopsy is usually indicated for diagnosis. The average size of HSVN is 2 cm^2^, and the size range is between 1 cm and 15.9 cm as reported by Walcott-Sapp et al. [4]. In all known instances, the nodule presented in a background of liver disease [2].

The immunohistochemical stains for vascular markers CD34, CD31, and FLI-1 are uniformly positive in HSVN [1, 2]. The proliferation rate by Ki-67 is low (mean: 3.7%), which is the best tool to differentiate this lesion from angiosarcoma, which in all cases is more than 10% [1, 2]. In addition to Ki-67, the immunohistochemical stains for p53 and c-Myc can be helpful in the diagnosis. Strong p53 and diffuse c-Myc stains are present in angiosarcoma and are absent in HSVN [1].

The differential diagnosis of HSVN includes cavernous hemangioma, anastomosing hemangioma, epithelioid hemangioendothelioma, and angiosarcoma. Among these vascular lesions, the histologic features of anastomosing hemangioma can be very similar to HSVN. Although most cases of anastomosing hemangioma are reported in the retroperitoneum, kidney, and ovary [5], it has rarely been described in the liver [5–8].

The distinction between these two entities is that the anastomosing hemangioma is well demarcated without liver infiltration. In addition, anastomosing hemangiomas usually have areas similar to cavernous hemangiomas, but this histologic finding is never reported in HSVN [6].

Molecular studies performed on HSVN cases have shown the presence of GNAQ and GNA14 mutations. These have not been reported in cavernous hemangioma or angiosarcomas [2, 9].

The rarity of this neoplasm means it has likely been underdiagnosed in the past. HSVN should enter the differential diagnosis of the pathologist whenever a hepatic vascular lesion is encountered, particularly if it has an infiltrative margin. The diagnosis of HSVN can then be confirmed or ruled out using the immunohistochemical stains discussed above. Although there have been no reported cases with recurrence or metastasis, the course of the disease is not well understood, so close follow-up is recommended [2, 10]. If the tumor is diagnosed on needle liver biopsy, complete resection is recommended [1]. In our case, the HSVN was found in the explant liver and there is no report of HSVN prognosis in patients receiving immunosuppressive medications.

## 4. Conclusion

We report a case of HSVN incidentally found in an explant liver; the lesion was not seen in radiographic images prior to the surgery. The awareness of this neoplasm is necessary to make an accurate diagnosis and differentiate it from other liver lesions especially angiosarcoma. As the number of reported patients is low and there are not enough data about the long-term prognosis, resection and close follow-up are recommended.

## Data Availability

The recut of the pathology slides and additional pictures of the slides are available from the corresponding author upon request.
